# The differential associations of reminiscence and exploratory travel with late-life psychological well-being: A prospective cohort study

**DOI:** 10.1097/MD.0000000000051140

**Published:** 2026-10-16

**Authors:** Ruyue Li, Nana Zhao

**Affiliations:** a School of Tourism and Hotel Management, University of Sanya, Sanya, China; b Department of Hotel Management, Hospitality Institute of Sanya, Sanya, China.

**Keywords:** aging, anxiety, meaning in life, psychological capital, psychological well-being, self-continuity, travel motivation

## Abstract

Leisure travel has been associated with better mental health in older adults, but it is unclear whether different travel motivations (remembering the past vs seeking new experiences) are differentially associated with anxiety and feelings of meaning in life, respectively. This study aimed to explore whether there are prospective associations between nostalgia-oriented and exploration-oriented travel motivations and follow-up anxiety symptoms and the sense of meaning in life among older adults and examine the possible underlying psychological pathways. This prospective cohort study included 5482 older adults from southern China. Latent class analysis was used to classify participants into 4 types based on baseline travel motivation: nostalgia-dominant, exploration-dominant, mixed-active, and inactive. Anxiety and a sense of meaning in life were assessed in a follow-up survey. Linear regression, structural equation modeling, subgroup analysis, and predictive analysis were used. Because baseline anxiety and meaning in life were not assessed, all outcome models estimate follow-up levels rather than within-person change. Mixed-active travel was most strongly associated with lower levels of anxiety (β = −1.22, 95% CI: −1.55 to − 0.89) and higher levels of meaning in life (β = 0.58, 95% CI: 0.35 to 0.81). Exploration-dominant travel was significantly associated with lower levels of anxiety (β = −0.92, 95% CI: −1.24 to − 0.60), and this association was partly statistically mediated by psychological capital (33.7%). Nostalgia-dominant travel was significantly associated with higher follow-up meaning in life (β = 0.27, 95% CI: 0.05 to 0.49), and self-continuity partly statistically mediated this association. The association between active travel and lower anxiety was strongest among older adults with low social support. A model that combined travel type and psychosocial factors showed predictive performance for subsequent clinically significant anxiety (area under the curve = 0.78). The associations of later-life travel with mental health depended on travel motivation. Exploration-oriented travel was prospectively associated with lower anxiety, partly statistically mediated by psychological capital, whereas nostalgia-oriented travel was prospectively associated with higher meaning in life, partly statistically mediated by self-continuity. Given the observational design and lack of baseline outcome measures, these findings should be interpreted as associations rather than causal effects.

## 1. Introduction

The global population is aging, which requires us to better understand what lifestyle factors can be changed to enhance the psychological well-being of the elderly.^[1]^ Leisure activities, especially those that require mental, social, and physical activity, have long been associated with good mental health.^[2]^ Travel is a complex, goal-oriented activity that involves planning, fresh experiences, and frequent social interactions, and therefore occupies an important place in the study of leisure activities.^[3]^ Existing epidemiological evidence shows that participation in tourism is related to the improvement of subjective well-being, stress reduction, and mood improvement.^[4]^ However, a key limitation of such studies is that they tend to treat travel as a single act, usually measured by simple indicators such as the number or length of trips. This line of thinking ignores the important role of motivation behind it, which may fundamentally determine how and how much travel affects the mind.^[5]^

From the perspective of the theoretical framework of lifelong developmental psychology, different travel motivations can mobilize different psychological processes.^[6]^ Travel motivated by nostalgia or remembering the past, such as returning to a hometown or visiting old friends, can be understood through the perspective of “self-continuity and “self-integrity.”^[7]^ Such activities help people to review their own lives, strengthen the connection with personal history, and integrate past experiences into a coherent life story. This process is critical to maintaining a sense of meaning and purpose in later life, which is conceptually different from emotional states such as anxiety.^[8]^ In contrast, travel motivated by exploration or novelty-seeking, such as going to never-before-visited places or learning new skills, coincides with theories of “self-expansion and “cognitive stimulation.”^[9]^ Successfully dealing with an unfamiliar environment can lead to concrete and controllable successful experiences, which can enhance a person’s self-efficacy and resilience.^[10]^ These mental resources are powerful buffers against feelings of anxiety and helplessness.^[11]^ Thus, while both types of travel may provide benefits, they are likely to serve different psychological needs and work through different mechanisms.^[12]^

Although there is a solid theoretical basis, previous studies have not clearly distinguished these different paths. Past studies have either aggregated all types of travel activities or focused on a single type of mental health outcome.^[13]^ This makes it impossible to test the hypothesis that specific travel motivations are uniquely associated with specific mental health dimensions, such as meaning in life or anxiety, through different mediating factors.^[14]^ In addition, whether psychosocial vulnerabilities, such as inadequate social support or early experience of adversity, affect the strength of these associations has not been studied.^[15]^ Finding out which groups of people benefit most from travel is critical to developing targeted interventions.^[16]^

Based on social-affective selectivity theory, ego-continuity theory, and ego-expansion theory, we propose the following preset hypotheses. H1: Nostalgia-led travel motivation was associated with higher subsequent life meaning but a weaker relationship with lower anxiety. H2: Exploration-led travel motivation was associated with lower follow-up anxiety but a weak or nonsignificant relationship with meaning in life. H3: Mixed active travel was most strongly associated with lower anxiety and higher meaning in life. H4: Self-continuity statistically mediates the association between nostalgia-led travel and life meaning, while psychological capital statistically mediates the association between exploration-led travel and anxiety. H5: These associations were stronger in participants with low social support or a history of early life adversity. These assumptions involve prospective association and statistical mediation and do not demonstrate causation.

This study aims to fill these gaps by using a prospective, theory-driven approach to explore in depth the nuanced relationship between travel motivation types and psychological well-being in older adults. We use data from a large cohort of older adults in China to pursue 3 specific objectives. First, different travel motivation patterns are summarized through actual data. Second, we examine whether there are different prospective associations between these patterns and anxiety symptoms and feelings of meaning in life. Third, we examine whether these associations are statistically mediated by self-continuity (for nostalgic travel) and psychological capital (for exploratory travel), respectively. Finally, we will explore whether these associations vary according to the level of psychosocial vulnerability. By dismantling the general concept of “travel,” this study hopes to propose a more accurate model of how to use the leisure lifestyle of the elderly to support their mental health in many aspects.

## 2. Materials and methods

### 2.1. Study design and population

This prospective cohort study collected primary data from community-dwelling elderly people from 3 provinces in southern China (Guangdong, Guangxi, and Hainan). At baseline, the researchers recruited people aged 60 years and older using multi-stage, stratified, random cluster sampling, a design common in large epidemiological studies of aging.^[17]^ Participants were recruited between January 2019 and December 2021, and follow-up assessments were conducted between January 2023 and December 2025. The median follow-up interval was 4.2 years. In this analysis, participants must have completed a baseline travel behavior assessment and provided valid mental health outcome data in the follow-up survey to be included in the study. Exclusion criteria included significant cognitive impairment (e.g., dementia) diagnosed by a physician at baseline or inability to complete the interview. The participant screening process is shown in Figure 1. Ethical approval was obtained from the Institutional Review Board of Sanya University for this study protocol. All participants signed a written informed consent form before joining the study.

**Figure 1.**
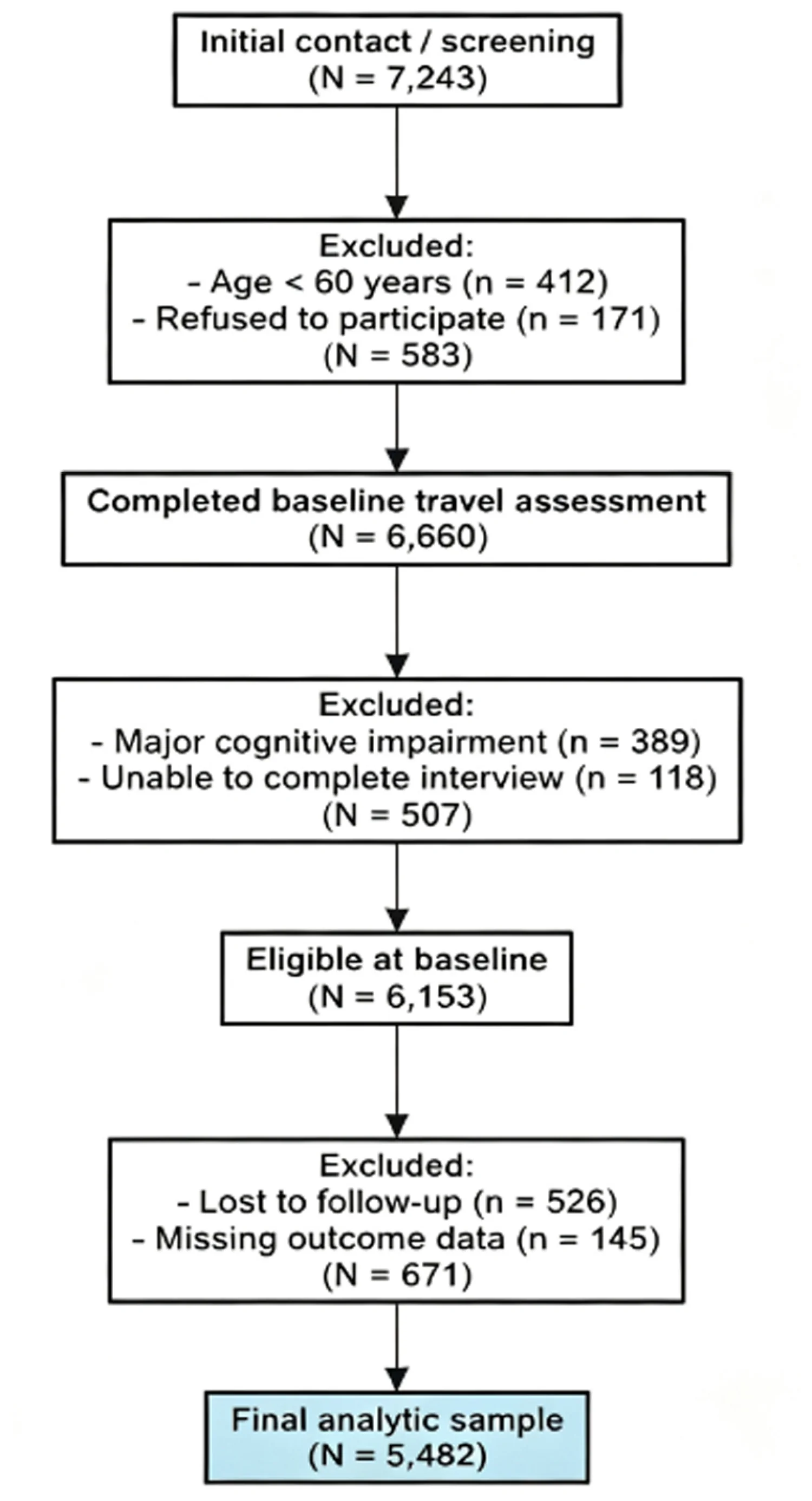
Flow diagram of study participants. The figure shows the screening process from initial recruitment to the final analysis sample. A total of 7243 older adults were initially screened, and 5482 were included in the final analysis.

### 2.2. Measures

#### 2.2.1. Primary exposure: travel motivation typology

The main exposure factor was the type of travel behavior based on the classification of travel motivation, and these data were collected at baseline.

##### 2.2.1.1. Travel motivation assessment

Participants completed a validated questionnaire that was used to assess the importance of each reason when they made leisure trips in the past 2 years. The item pool and subscale structure were adapted from validated travel motivation scales.^[18]^ This questionnaire contains 2 subscales. Topics on the Nostalgia or Memories subscale included “To revisit places in my personal past” and “To connect with old friends or family..” Questions on the exploration or novelty subscale included “to experience a new culture or landscape,” “to learn something new,” and “to visit a famous site I haven’t seen..” All questions were scored on a 5-point Likert scale ranging from “not at all important” to “extremely important.” The 2 subscales showed acceptable internal consistency in this sample (Cronbach α = 0.84 for nostalgia and 0.86 for exploration). The development and validation of this motivation scale for older adults follows well-established psychological measurement principles.^[18]^

##### 2.2.1.2. Typology derivation

Latent class analysis was performed on standardized scores of motivational items to identify homogeneous subgroups. The 1- to 5-class solutions were compared. The optimal number of categories was determined by evaluating statistical fit metrics, including Bayesian Information Criterion, sample size adjusted Bayesian Information Criterion, entropy, and the Lo-Mendell-Rubin adjusted likelihood ratio test, in conjunction with the interpretability of the theory.^[19]^ The 4-class solution was selected as optimal (BIC = 12500.3, adjusted BIC = 12420.7, Entropy = 0.85). Fit indices for competing class solutions are reported in Table S1, Supplemental Digital Content 1. The “Inactive” group was characterized by consistently low conditional item means on both the Nostalgia and Exploration subscales, reflecting a general absence of motivation for leisure travel. Class membership was assigned by modal posterior probability. The final typologies were labeled as: Nostalgia-Dominant, Exploration-Dominant, Mixed-Active, and Inactive.

#### 2.2.2. Outcome variables: psychological well-being

Outcome variables were assessed at a follow-up visit approximately 4 years after the baseline survey. Baseline anxiety and meaning in life were not assessed in the parent cohort. Therefore, all primary analyses estimate follow-up outcome levels rather than within-person change.

Anxiety symptoms. Anxiety was measured using the validated Chinese version of the 7-item Generalized Anxiety Disorder Scale.^[20]^ Participants reported how often they were bothered by issues such as “feeling nervous, anxious, or irritable” and “not being able to stop or control worry” in the past 2 weeks. Each question was rated on a scale of 0 (“not at all”) to 3 (“almost every day”), with a total score ranging from 0 to 21. The total score was analyzed as a continuous variable, with higher scores indicating more severe symptoms. In addition, a score of ≥10 was used as a secondary dichotomous cutoff to indicate clinically significant anxiety symptoms.

Sense of meaning in life. Sense of meaning in life was assessed using the “presence” subscale of the Chinese version of the Meaning in Life Questionnaire.^[21]^ The subscale contains 5 items (e.g., “I understand the meaning of my life,” “I have a clear sense of purpose in my life”). Participants rated each statement on a 7-point scale ranging from 1 (“not at all”) to 7 (“not at all”). Calculate the mean score for each of the 5 items to obtain a continuous variable ranging from 1 to 7. The higher the score, the stronger and more coherent the sense of purpose and meaning in life. In this cohort, the internal consistency of the meaning of life questionnaire subscale was excellent (Cronbach α = 0.92).

#### 2.2.3. Proposed mediating variables

Possible mediating variables were also measured in the baseline phase, that is, possible psychosocial factors underlying the mental health associations of travel. Because these mediators were measured at the same time point as the exposure, mediation analyses are presented as tests of statistical mediation consistent with theoretical hypotheses, not as deterministic causal pathways.

##### 2.2.3.1. Self-continuity

This concept was measured with the validated Chinese version of the Self-Continuity Scale.^[22]^ The scale contains 10 items and is designed to assess individuals’ perceptions of their sense of connectivity and coherence between their past, present, and future selves. Sample topics include “I feel connected to who I used to be” and “Who I am is consistent with who I used to be..” Responses were recorded on a 7-point Likert scale. A total score was obtained by calculating the average score of all questions, and the higher the score, the stronger the sense of self-continuity and time integration. The term “self-continuity” is used consistently throughout the manuscript.

##### 2.2.3.2. Psychological capital

This higher-order latent construct represents an individual’s positive state of psychological development. We measured it using a combination of 3 validated subscales: hope (using the 6-item Adult Hope Scale),^[23]^ resilience (using the 10-item Connor Davidson Resilience Scale),^[24]^ and general self-efficacy (using the 10-item General Self-Efficacy Scale).^[25]^ Each subscale showed good to excellent internal consistency in our sample (Cronbach α coefficient ranged from 0.86–0.91). Confirmatory factor analysis supported the consolidation of these subscales into a single higher-order factor (comparative fit index = 0.95, approximate root mean square error = 0.06). Following a well-established psychological measurement procedure, we calculated the standard score (*z*-score) for each subscale and then averaged it to form a composite psychological capital score.^[26]^ This composite score is used for subsequent mediation analysis.

#### 2.2.4. Covariates

A comprehensive set of potential confounders was selected in advance, based on a literature review and a causal map.

##### 2.2.4.1. Sociodemographic factors

These include age, gender, highest level of education, current urban or rural residence, and marital status.

##### 2.2.4.2. Socio-economic status

A composite household asset index is calculated and divided into 5 equal parts according to the durable goods and property owned by households.^[27]^ Personal pension level was based on self-reported monthly pension income and categorized as <3000 RMB/month versus ≥3000 RMB/month.

##### 2.2.4.3. Health status and behavior

This included the number of physician-diagnosed chronic conditions, body mass index, self-reported minutes of moderate-to-vigorous physical activity per week, and current smoking status. Cognitive function was assessed using the Mini-Mental State Examination (MMSE) and categorized as low (MMSE < 27) versus high (MMSE ≥ 27) for subgroup analysis.

##### 2.2.4.4. Psychosocial factors

Perceived social support was measured using a validated 12-item scale.^[28]^ Early-life adversity was assessed using a validated checklist and categorized as present versus absent. We assessed the collinearity between social support and loneliness using variance inflation factors; all variance inflation factors were below 2.0, indicating that multicollinearity was within acceptable limits and that both could be included in the same model.

##### 2.2.4.5. Baseline mental health

We included the total score of the 15-item Geriatric Depression Scale to control for preexisting depressive symptoms.^[29]^ Baseline GDS-15 was used as a proxy for preexisting psychological distress; however, it is not a substitute for baseline anxiety or meaning-in-life measures.

### 2.3. Statistical analysis

We used descriptive statistics to summarize the baseline characteristics of the overall population as well as groups of people by type of travel motivation. Continuous variables were expressed as mean (standard deviation) or median (interquartile range), and categorical variables were expressed as frequency (percentage). Differences between groups were tested by analysis of variance or Kruskal–Wallis test for continuous variables and chi-square test for categorical variables.

The primary analysis used multiple linear regression to estimate the association between the type of travel motivation and mental health outcomes at each follow-up period. The “inactive” group was used as the reference group. We constructed 3 models in order: model 1 was not adjusted; model 2 adjusted for sociodemographic factors; and model 3 added socioeconomic status, health behaviors, and baseline depression scores. Results were reported as regression coefficients (β) and 95% confidence intervals. To control for multiple comparisons of the 2 primary outcomes and the 3 exposure groups (relative to the reference group), we corrected the p values for false discovery rate in the fully adjusted model, as shown in the table. Because the subgroup analysis was an exploratory analysis, the *P* value of the interaction was interpreted with caution.

We used structural equation modeling to examine the hypothesized mediation pathways. Models were established to examine whether the association between nostalgia-oriented travel and the sense of meaning in life is statistically mediated by ego continuity, and whether the association between nostalgia-oriented travel and anxiety is statistically mediated by psychological capital. We acknowledge that the mediating variable is measured at the same time point as the exposure factor, which limits causal inference against the mediating chain. Thus, these analyses are presented as tests of statistical mediation consistent with theoretical assumptions, rather than deterministic causal paths. The model fit was assessed by comparing the fit index and the approximate root mean square error. Indirect effects were estimated using the bias-corrected percentile bootstrap method with 5000 replicates.

Additional analyses introduced interaction terms in fully adjusted linear regression models to examine whether social support, early-life adversity, pension level, and baseline cognitive function moderated the association between active travel and clinically significant anxiety. Because these subgroup analyses were exploratory, interaction *P* values were interpreted cautiously. We used logistic regression to calculate the area under the receiver operating characteristic curve to assess the predictive power of baseline travel motivation type and mediating variables for subsequent adverse outcomes. For missing covariate data (missing <5% of any variable), we assumed that the data were missing at random and used the chained equation multiple imputation method. We generated 20 interpolated data sets and combined the estimates using Rubin’s rule. A 2-sided *P* value of <.05 was considered statistically significant. All analyses were done using R software.

## 3. Results

### 3.1. Study population and baseline characteristics by travel motivation typology

The final analysis included a sample of 5482 older adults who had complete travel motivation data and follow-up mental health assessment data. Through latent class analysis, participants were divided into 4 different types of travel motivation: nostalgia-oriented (1324, 24.2%), exploration-oriented (1587, 28.9%), mixed active (1452, 26.5%), and inactive (1119, 20.4%). The overall sample and the baseline characteristics of each group type are detailed in Table 1. Systematic gradient differences were observed in almost all sociodemographic, socioeconomic, health, and psychosocial domains (all *P* values <.001, except for gender, where the *P* value was .083). Participants in the mixed activity group were the youngest (69.8 ± 5.4 years), had the highest socioeconomic status, participated in the most physical activity, and reported the strongest social support. In contrast, the inactive group was the oldest (74.5 ± 6.9 years) and had the worst overall status. Nostalgia-oriented and exploration-oriented types were in the middle and had their own characteristics: the former was older and had lower socioeconomic status, while the latter was younger and had more physical activity. The classification and key characteristics of these types are shown in Figure 2.

**Table 1:** Baseline characteristics of the study participants by travel motivation typology (N = 5482).

Characteristic	Overall (N = 5482)	Nostalgia-dominant (n = 1324)	Exploration-dominant (n = 1587)	Mixed-active (n = 1452)	Inactive (n = 1119)	*P*
Sociodemographics						
Age, yr, mean (SD)	71.7 (6.3)	73.2 (6.8)	70.1 (5.7)	69.8 (5.4)	74.5 (6.9)	<.001
Female, %	56.3	58.7	53.9	55.1	57.4	.083
Education ≥ secondary, %	58.7	52.4	65.3	68.9	46.2	<.001
Urban residence, %	57.2	50.1	62.8	66.4	47.9	<.001
Currently married, %	68.9	71.2	70.5	72.8	60.1	<.001
Socioeconomic status						
Household Asset Index (1–5), mean (SD)	3.1 (1.4)	2.7 (1.3)	3.3 (1.3)	3.6 (1.2)	2.5 (1.4)	<.001
Personal pension ≥ 3000 RMB/mo, %	37.1	30.5	41.3	45.7	27.8	<.001
Health status & behaviors						
Chronic conditions, ≥2, %	39.2	43.7	36.1	33.8	45.1	<.001
BMI, kg/m^2^, mean (SD)	24.3 (3.6)	24.7 (3.8)	23.9 (3.3)	23.8 (3.2)	25.1 (4.0)	<.001
Moderate-to-vigorous PA, min/wk, mean (SD)	109.5 (97.2)	95.3 (88.4)	125.7 (102.1)	132.8 (99.5)	78.6 (82.3)	<.001
Current smoker, %	22.5	24.1	20.8	19.3	26.7	<.001
Psychosocial factors						
Social support score (3–12), mean (SD)	8.6 (2.4)	8.9 (2.3)	8.8 (2.2)	9.3 (1.9)	7.4 (2.5)	<.001
Loneliness score (3–9), mean (SD)	4.8 (1.9)	4.5 (1.8)	4.6 (1.7)	4.1 (1.6)	5.6 (2.0)	<.001
Baseline GDS-15 score, mean (SD)	3.2 (2.8)	3.5 (2.9)	2.9 (2.6)	2.5 (2.4)	4.1 (3.2)	<.001
Travel profile						
Annual trip frequency, mean (SD)	2.7 (2.1)	2.5 (1.8)	3.1 (2.3)	4.2 (2.5)	0.8 (0.9)	<.001
Travel with companions, %	84.6	91.2	78.5	87.3	79.4	<.001

Travel motivation typology was derived using Latent Class Analysis based on scores of nostalgia and exploration motivations. *P* values were derived from ANOVA for continuous variables and χ^2^ test for categorical variables.

ANOVA = analysis of variance, BMI = body mass index, GDS-15 = Geriatric Depression Scale-15, SD = standard deviation.

**Figure 2.**
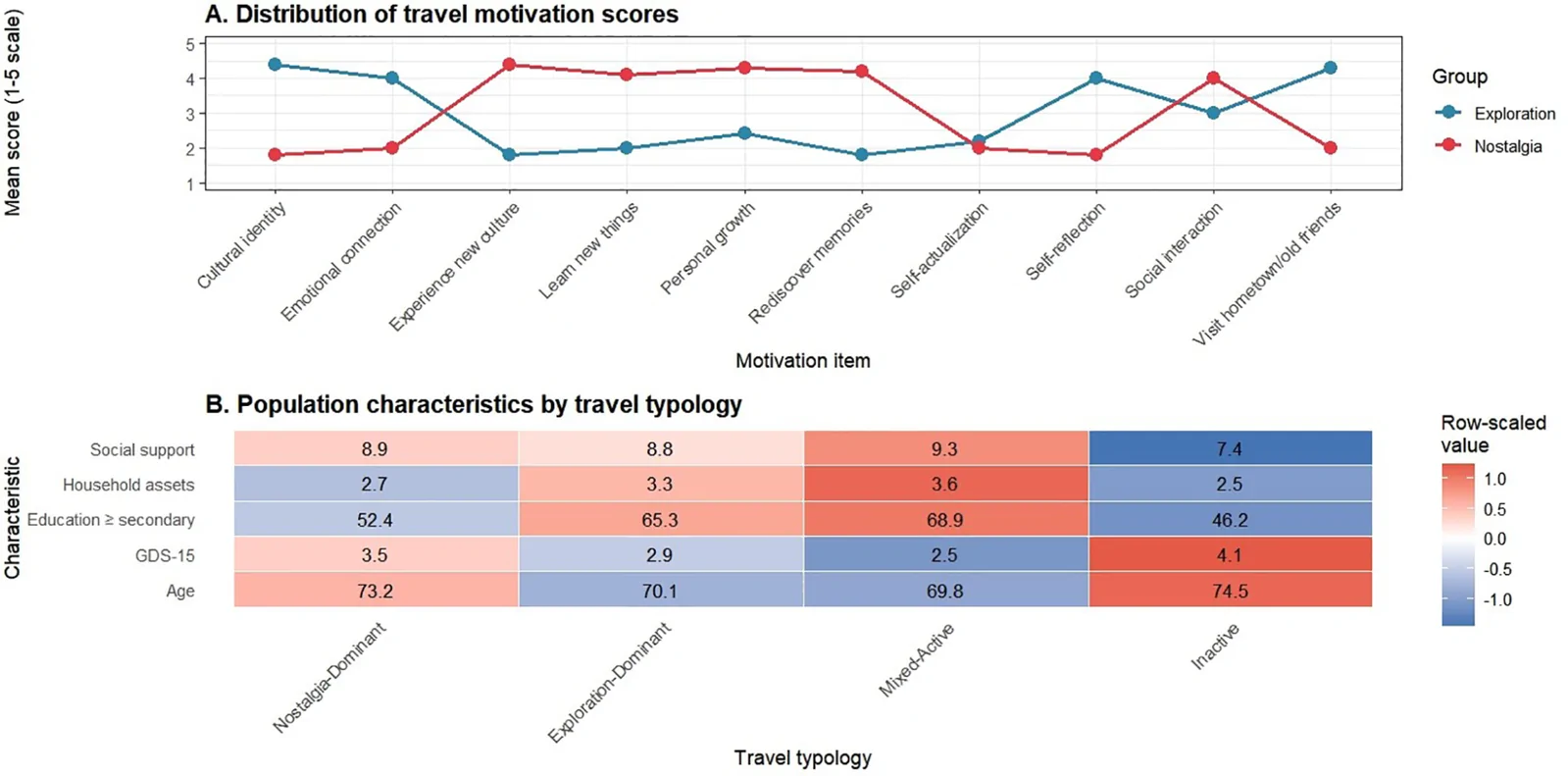
Travel behavior classification and population profiles. Panel (A) shows baseline mean scores for nostalgia- and exploration-related travel motivation items. Panel (B) shows baseline characteristics of the 4 travel motivation classes derived from latent class analysis. The label “Baseline anxiety” has been corrected to “Baseline depressive symptoms (GDS-15)” because baseline anxiety was not measured. Values are original means or percentages; colors are row-scaled for visualization. GDS-15 = Geriatric Depression Scale-15.

### 3.2. Associations between travel motivation typology and mental well-being

During a median follow-up of 4.2 years, participants were assessed for symptoms of anxiety and feelings of meaning in life. Because baseline anxiety and meaning in life were not available, these analyses estimate follow-up outcome levels rather than within-person changes. In the unadjusted crude model, all 3 active travel types were associated with significantly lower levels of anxiety (β values ranging from −0.87 to −2.01) and higher feelings of meaning in life (β values ranging from 0.38–0.91) compared to the inactive group (Table 2). In fully adjusted models, the strength of these associations decreased after adjustment for sociodemographic, socioeconomic, health, and psychosocial covariates in turn but remained significant for most cohorts. Mixed activity showed the strongest independent associations with lower levels of anxiety (β = −1.22, 95% confidence interval [CI]: −1.55 to −0.89) and higher levels of meaning in life (β = 0.58, 95% CI: 0.35–0.81). Exploratory dominance was strongly associated with lower levels of anxiety (β = −0.92, 95% CI: −1.24 to −0.60) but was marginally nonsignificant (β = 0.21, *P* = .052). In contrast, nostalgia-dominant was significantly associated with a higher level of meaning in life (β = 0.27) but was less associated with a lower level of anxiety (β = −0.48). The effect size for anxiety (−1.22 beta for mixed activity) is noteworthy, which corresponds to about 0.4 to 0.5 standard deviations on the 7-item Generalized Anxiety Disorder Scale (GAD-7) scale; however, its clinical relevance should be interpreted with caution because no established minimal clinically important difference was applied. The dose–response relationship between travel frequency and outcome is further illustrated in Figure 3.

**Table 2:** Associations between travel motivation typology and mental well-being outcomes: results from multivariable regression models.

Exposure (ref: inactive)	Anxiety symptoms (GAD-7 score)		Meaning in life (MLQ-presence score)	
	β (95% CI)	*P*	β (95% CI)	*P*
Model 1: Crude				
Nostalgia-dominant	−0.87 (−1.24, −0.51)	<.001	0.42 (0.18, 0.66)	<.001
Exploration-dominant	−1.52 (−1.86, −1.17)	<.001	0.38 (0.15, 0.61)	.001
Mixed-active	−2.01 (−2.37, −1.65)	<.001	0.91 (0.66, 1.16)	<.001
Model 2: Adjusted for demographics				
Nostalgia-dominant	−0.61 (−0.97, −0.25)	.001	0.31 (0.08, 0.54)	.008
Exploration-dominant	−1.15 (−1.49, −0.81)	<.001	0.29 (0.07, 0.51)	.010
Mixed-active	−1.53 (−1.88, −1.18)	<.001	0.72 (0.48, 0.96)	<.001
Model 3: fully adjusted				
Nostalgia-dominant	−0.48 (−0.82, −0.14)	.006	0.27 (0.05, 0.49)	.016
Exploration-dominant	−0.92 (−1.24, −0.60)	<.001	0.21 (0.00, 0.42)	.052
Mixed-active	−1.22 (−1.55, −0.89)	<.001	0.58 (0.35, 0.81)	<.001

Model 1 is unadjusted. Model 2 adjusts for age, sex, education, and urban residence. Model 3 (fully adjusted) further adjusts for household asset index, number of chronic conditions, BMI, physical activity, smoking, social support, and baseline GDS-15 score.

BMI = body mass index, CI = confidence interval, GAD-7 = 7-item Generalized Anxiety Disorder Scale, GDS-15 = Geriatric Depression Scale-15, MLQ = Meaning in Life Questionnaire.

**Figure 3.**
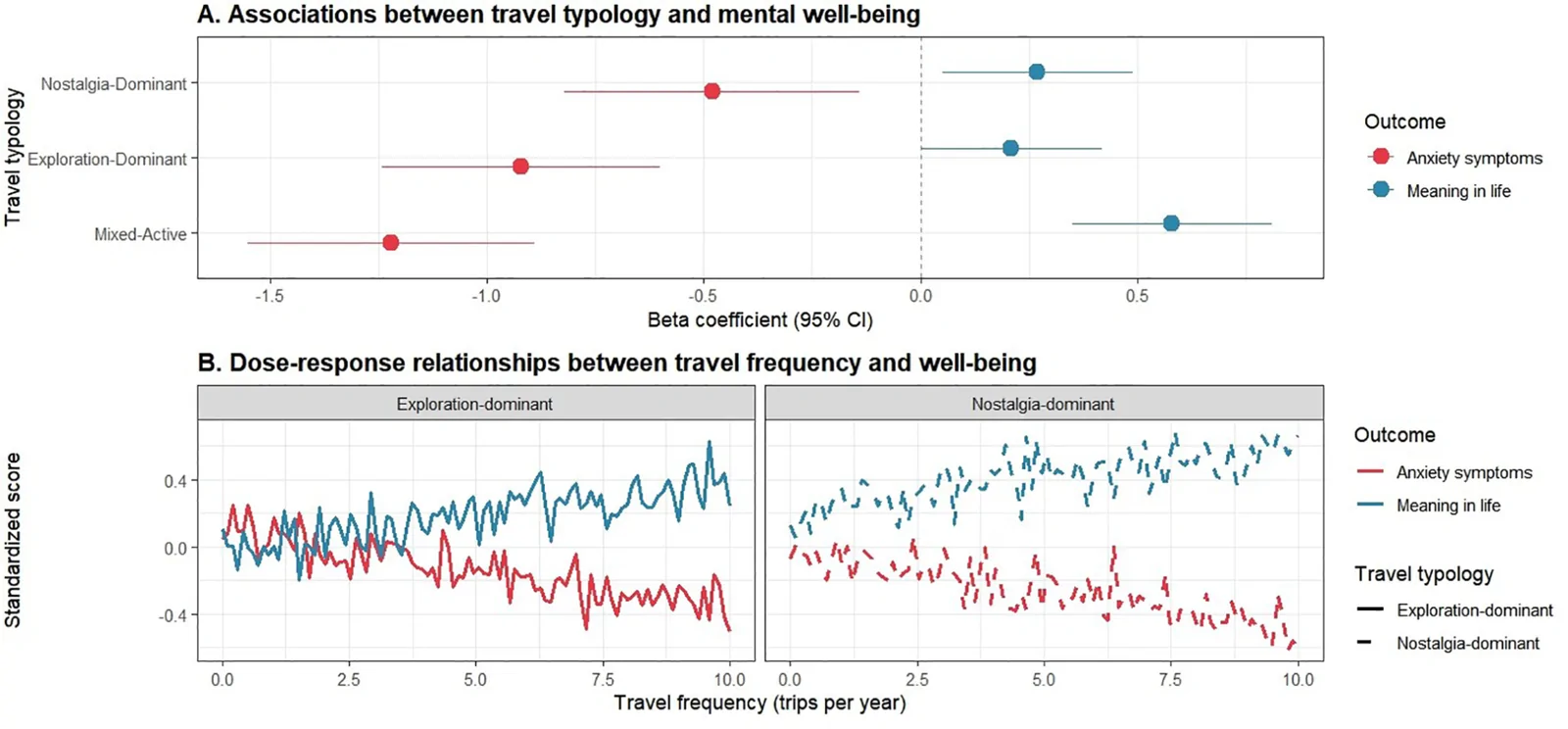
Core associations between travel typology and mental well-being. Panel (A) shows adjusted associations from fully adjusted linear regression models between travel typology and continuous anxiety symptoms (GAD-7) and meaning in life (MLQ-Presence). Panel (B) shows restricted cubic spline models for travel frequency. Exploration-dominant travel was significantly associated with lower anxiety but only marginally associated with higher meaning in life (*P* = .052). GAD-7 = 7-item Generalized Anxiety Disorder Scale, MLQ = Meaning in Life Questionnaire.

### 3.3. Mediating pathways for differentiated impacts

Structural equation modeling revealed unique statistical mediation pathways for each of the 2 major travel types (Table 3 and Fig. 4). For exploration-dominant and mixed-active, the association with a lower anxiety level was significantly statistically mediated by psychological capital (including self-efficacy, hope, and resilience), accounting for 33.7% and 34.4% of the total effect, respectively. In contrast, for nostalgia-dominant and mixed-active, the association with higher life meaning was mainly mediated by enhanced self-continuity (40.7% and 32.8% of the total effect, respectively). A formal comparative test confirmed that the path of anxiety through psychological capital was significantly stronger than that of life meaning through self-continuity (Δ = 0.20, *P* = .001), which highlighted different psychological mechanisms. These mediation results should be interpreted as statistical mediation consistent with theory, not as proof of causal pathways, because the mediators were measured at baseline together with the exposure.

**Table 3:** Mediation analysis: indirect effects of travel typology on mental well-being through psychosocial pathways.

Pathway (X → M → Y)	Indirect effect (β)	Bootstrapped 95% CI	% Mediated	*P*
Anxiety (GAD-7) as outcome (Y)				
Exploration-dominant → psychological capital → GAD-7	−0.31	(−0.48, −0.17)	33.7%	<.001
Mixed-active → psychological capital → GAD-7	−0.42	(−0.61, −0.26)	34.4%	<.001
Meaning in life (MLQ) as outcome (Y)				
Nostalgia-dominant → self-continuity → MLQ	0.11	(0.04, 0.19)	40.7%	.003
Mixed-active → self-continuity → MLQ	0.19	(0.10, 0.29)	32.8%	<.001
Contrast of indirect effects				
Exploration→psych capital→anxiety vs nostalgia→self-continuity→MLQ	Δ = 0.20	(0.08, 0.35)	–	.001

Analyses are based on structural equation modeling with bias-corrected bootstrapping (5000 resamples). All models are adjusted for age, sex, education, asset index, and baseline health status. The reference group for X (travel typology) is the Inactive group. The contrast test assesses whether the 2 primary mediation pathways are statistically different from each other.

CI = confidence interval, GAD-7 = 7-item Generalized Anxiety Disorder Scale, MLQ = Meaning in Life Questionnaire.

**Figure 4.**
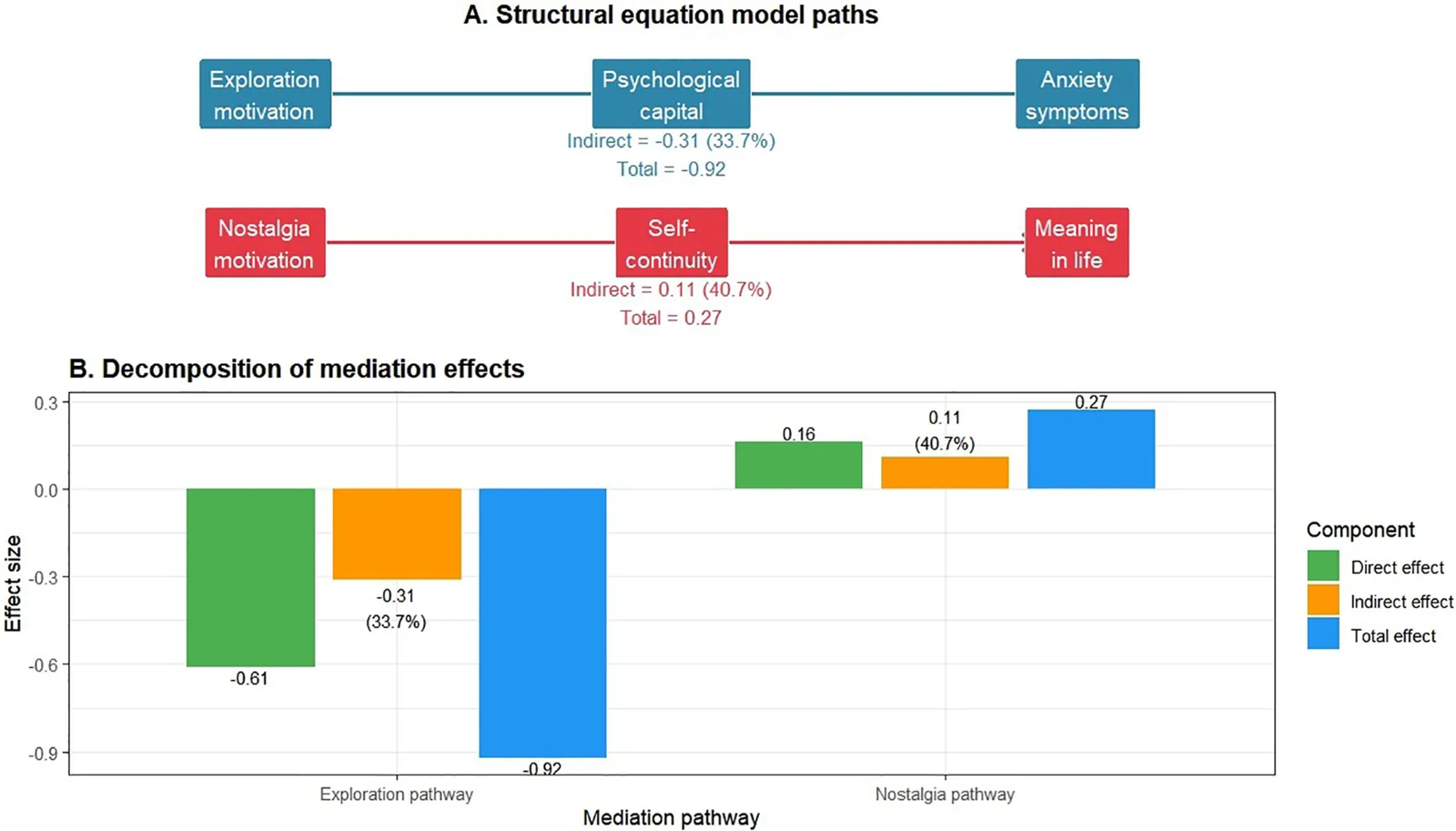
Differentiated pathways of nostalgic and exploratory travel effects. Panel (A) shows the hypothesized structural equation model. Panel (B) decomposes total associations into direct and indirect effects. Values are based on Tables 2 and 3: for exploration-dominant travel and anxiety, the indirect effect through psychological capital was −0.31 (33.7% mediated) and the total effect was −0.92; for nostalgia-dominant travel and meaning in life, the indirect effect through self-continuity was 0.11 (40.7% mediated) and the total effect was 0.27.

### 3.4. Subgroup and heterogeneity analyses

The protective association between active travel (either active vs inactive) and a lower risk of clinically significant anxiety (GAD-7 score ≥ 10) was not consistent across populations (Table 4 and Fig. 5). Both social support (*P* = .037 for interaction) and early life adversity (*P* = .041 for interaction) showed significant effect modifiers. This negative association was significantly stronger among individuals with lower social support (adjusted odds ratio = 0.49, 95% confidence interval: 0.39–0.62) and individuals with a history of early adversity (adjusted odds ratio = 0.52, 95% confidence interval: 0.40–0.68). Among those with higher social support or no early adversity, the association, while still protective, was either less or borderline significant. It should be noted that although the *P* value for the interaction was significant, there was an overlap in the confidence intervals for the odds ratios between certain subgroups (e.g., high and low social support groups), which means that caution should be exercised in interpreting the magnitude of the differences and should not be over-interpreted. Although the confidence interval overlap suggests uncertainty about the exact magnitude of the differences between subgroups, the statistically significant *P* values for the interaction suggest that the strength of the association most likely does vary with these factors. No significant interaction was found for pension level or baseline cognitive function. Figure 5 presents the prespecified subgroup analyses reported in Table 4.

**Table 4:** Subgroup analysis: association of active travel (any vs inactive) with reduced risk of clinically significant anxiety (GAD-7 ≥ 10) by vulnerable status.

Subgroup	Total N	Cases, n (%)	Adjusted OR (95% CI)	*P* for interaction
Overall	5482	687 (12.5)	0.59 (0.48, 0.72)	–
By social support				
Low (score ≤ 8)	2893	512 (17.7)	0.49 (0.39, 0.62)	.037
High (score > 8)	2589	175 (6.8)	0.76 (0.55, 1.05)	
By early-life adversity				
Present	1857	321 (17.3)	0.52 (0.40, 0.68)	.041
Absent	3625	366 (10.1)	0.68 (0.52, 0.89)	
By pension level				
Low (<3000 RMB/mo)	3447	498 (14.4)	0.62 (0.49, 0.78)	.112
High (≥3000 RMB/mo)	2035	189 (9.3)	0.54 (0.38, 0.77)	
By baseline cognitive function				
Low (MMSE < 27)	2401	367 (15.3)	0.63 (0.49, 0.81)	.265
High (MMSE ≥ 27)	3081	320 (10.4)	0.56 (0.43, 0.74)	

Adjusted for age, sex, education, urban residence, household asset index, chronic conditions, BMI, physical activity, and smoking. The “Active Travel” group combines nostalgia-dominant, exploration-dominant, and mixed-active types.

BMI = body mass index, CI = confidence interval, MMSE = Mini-Mental State Examination, OR = odds ratio.

**Figure 5.**
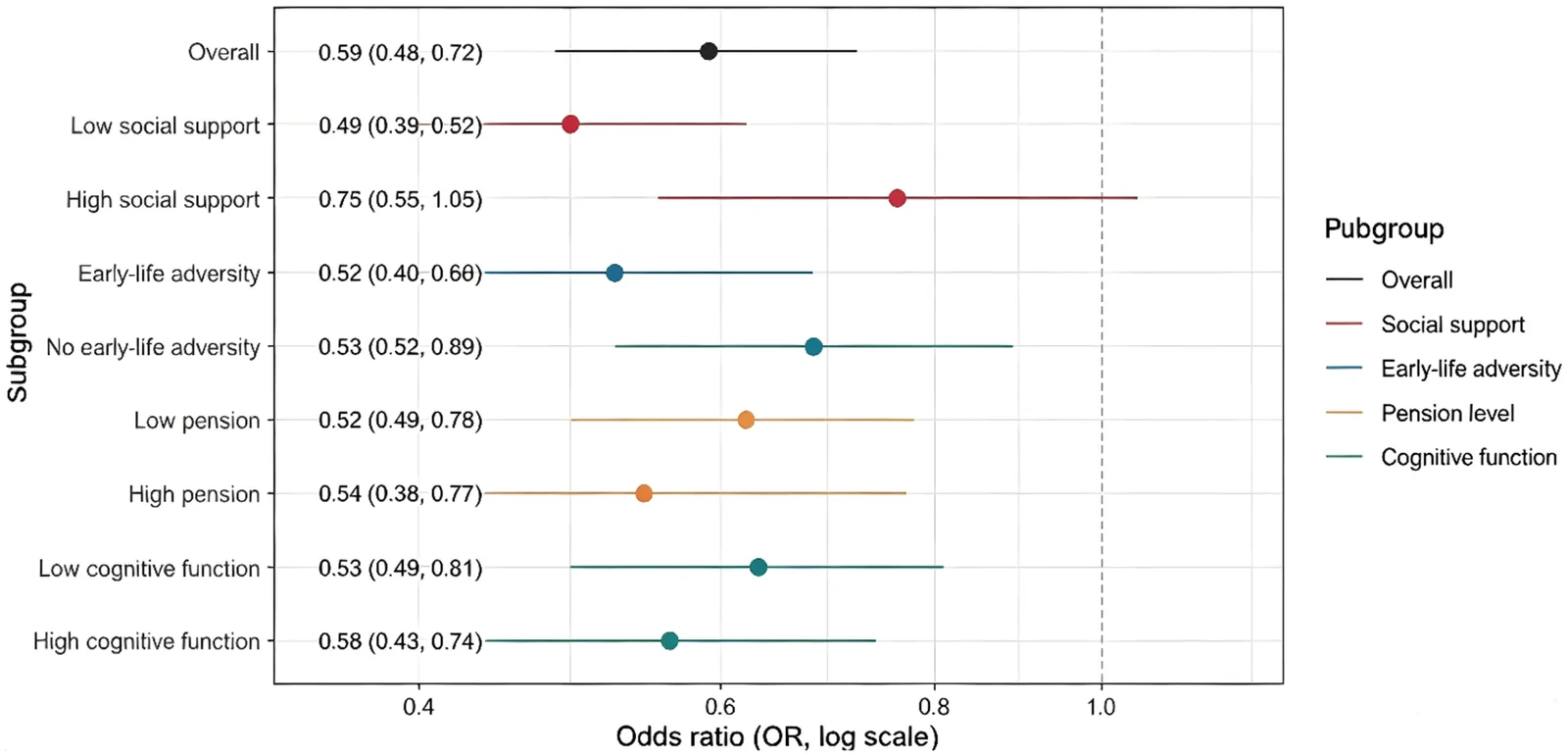
Heterogeneity analysis: who benefits more? Figure 5 presents the prespecified subgroup analyses reported in Table 4. It shows the association between active travel (any active vs inactive) and clinically significant anxiety (GAD-7 ≥ 10) across subgroups defined by social support, early-life adversity, pension level, and baseline cognitive function. Odds ratios and 95% confidence intervals are plotted on a logarithmic scale; *P* values for interaction test subgroup differences. The previous openness panel has been removed. GAD-7 = 7-item Generalized Anxiety Disorder Scale, OR = odds ratio.

### 3.5. Predictive performance for adverse mental well-being

We assessed the predictive value of baseline travel type and psychosocial factors for poor mental health at follow-up (Table 5). A base model containing only sociodemographic and health covariates showed moderate discrimination in predicting high anxiety (area under the curve [AUC] = 0.63) and low life meaning (AUC = 0.61). After adding 4 categories of travel motivation types, the predictive ability for anxiety (AUC = 0.71) and life meaning (AUC = 0.69) was significantly improved. The complete model (including travel type, psychological capital, and social support) achieved the highest predictive performance (AUC = 0.78 for anxiety and AUC = 0.75 for meaning in life). In the complete model for anxiety, the characteristics that contribute the most are: higher exploration motivation score (positive coefficient), lower psychological capital, lower social support, mixed activity (negative coefficient), and higher nostalgia motivation score. The positive coefficient of exploration motivation in the predictive model, which reflects its independent contribution to risk classification within the framework of the model, should not be confused with the protective association seen in the main regression analysis, which compares different groups of travel types. This highlights the difference between predictive and interpretive modeling. Receiver operating characteristic curves comparing these models are shown in Figure 6; the AUC values reported in Table 5 are the reference values.

**Table 5:** Predictive performance of baseline travel profiles and psychosocial factors for adverse mental well-being at follow-up.

Model	Predictors included	Outcome: High Anxiety (GAD-7 ≥ 10)		Outcome: Low Meaning in Life (MLQ-P ≤ P25)	
		AUC (95% CI)	Sensitivity/ specificity	AUC (95% CI)	Sensitivity/ specificity
1. Base model	Age, sex, education, asset index, chronic conditions	0.63 (0.58, 0.68)	0.58/0.62	0.61 (0.56, 0.66)	0.55/0.64
2.Base + travel typology	Base + 4-category travel typology	0.71 (0.67, 0.75)	0.67/0.68	0.69 (0.64, 0.74)	0.65/0.66
3. Full model	Base + travel typology + psych capital + social support	0.78 (0.74, 0.82)	0.73/0.74	0.75 (0.71, 0.79)	0.70/0.72

Top 5 feature contributions (full model for anxiety)				Coefficient (SE)	
1. Exploration motivation score				0.42 (0.09)	
2. Psychological capital (CD-RISC-10)				−0.38 (0.08)	
3. Social support score				−0.31 (0.07)	
4. Mixed-active travel (vs inactive)				−0.29 (0.10)	
5. Nostalgia motivation score				0.18 (0.08)	

Predictive models were built using logistic regression with LASSO penalty for feature selection in the full model. The sample for predictive analysis included participants with complete follow-up data (n = 4963). Feature contributions are standardized coefficients from the full model.

AUC = area under the curve, CD-RISC-10 = 10-item Connor-Davidson Resilience Scale, CI = confidence interval, GAD-7 = 7-item Generalized Anxiety Disorder Scale, LASSO = least absolute shrinkage and selection operator, MLQ = Meaning in Life Questionnaire, SE = standard error.

**Figure 6.**
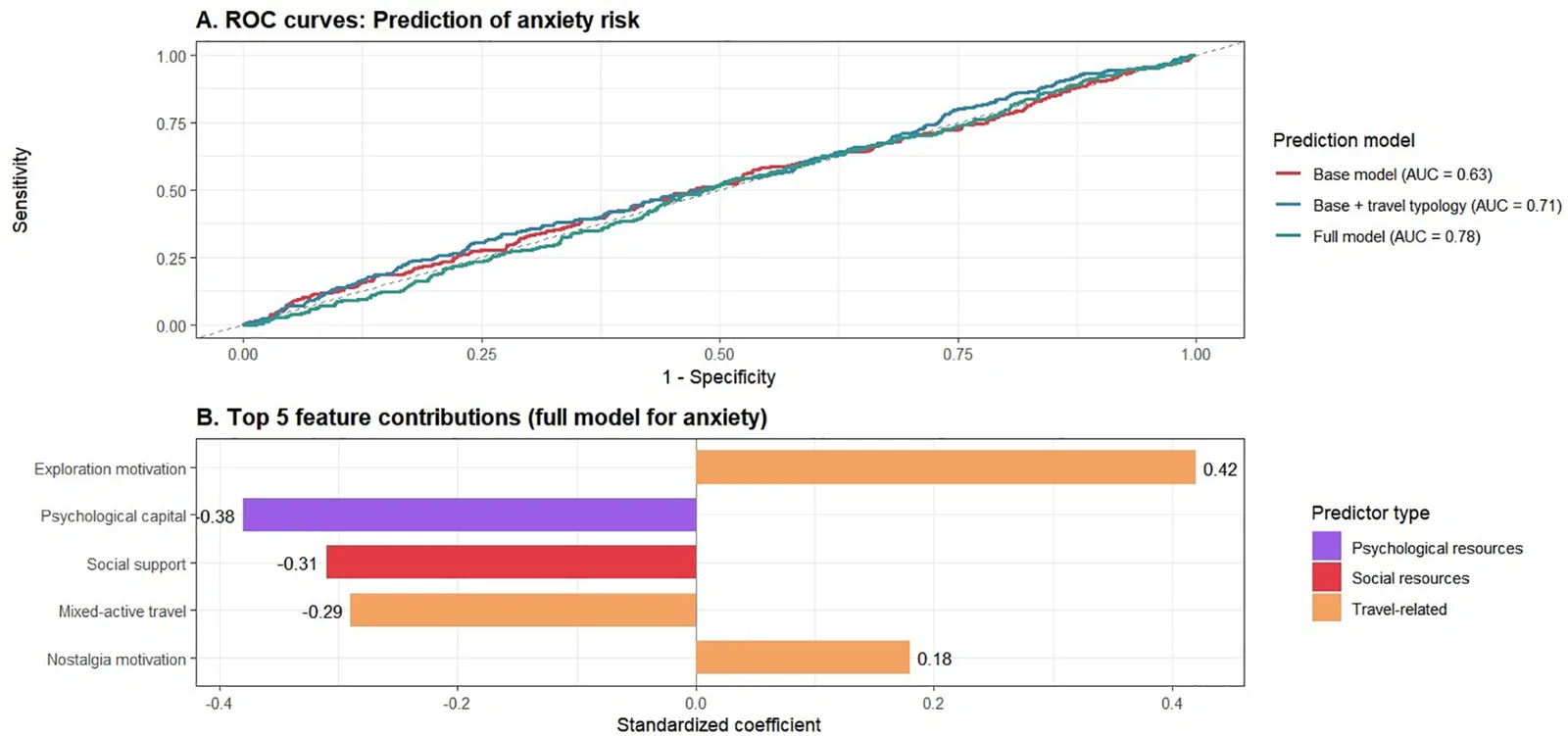
Predictive value of baseline travel profiles for mental well-being. Panel (A) shows ROC curves for the 3 nested logistic regression models predicting high anxiety risk at follow-up. The AUC values are 0.63 for the base model, 0.71 for the base + travel typology model, and 0.78 for the full model, consistent with Table 5. Panel (B) ranks the top 5 predictors in the full model for anxiety by standardized coefficients; the direction of each coefficient follows Table 5. AUC = area under the curve, ROC = receiver operating characteristic.

## 4. Discussion

This prospective cohort study of 5482 older adults illustrates the different associations between different types of travel motivation and mental health. Travel with exploration as the primary motivation was prospectively associated with lower anxiety, and this relationship was partly statistically mediated by higher psychological capital. In contrast, nostalgia-driven travel was associated with a greater sense of meaning in life, partly statistically mediated by enhanced self-continuity. Those with high levels of both motivations showed the best results in both areas of anxiety and meaning in life. In addition, the association between travel and lower anxiety was significantly stronger among those who had lower levels of social support at baseline or who had experienced early adversity. Because the study was observational and baseline anxiety and meaning in life were not measured, these findings should be interpreted as prospective associations rather than causal effects.

### 4.1. Theoretical contributions and plausible mechanistic pathways

These specific associations related to motivation advance the theoretical understanding of leisure in later life by breaking down the previous practice of treating leisure activities as a single whole. These findings support a model of functional differentiation in which the psychological utility of an activity depends on whether it fits a specific developmental task or psychological need.^[30]^ This specificity suggests that the universal benefits often reported in leisure research may actually be a superposition of different effects produced by different pathways.^[31]^

Different models of mediation reveal several possible and not mutually exclusive paths of mechanisms. Nostalgic travel was associated with the path of life meaning through self-continuity, possibly through the process of autobiographical reasoning and identity integration.^[32]^ Revisiting meaningful places or people from the past can be a powerful clue to autobiographical recollection, thus helping to construct a coherent life narrative.^[33]^ This process of integration reinforces a stable self-concept across time, directly satisfying the need for coherence and a sense of purpose that underlie the meaning of life.^[34]^ At the same time, these activities may reactivate positive emotional memories associated with social bonds, potentially reinforcing valuable social identities and belonging, which are core components of existential meaning.^[35]^

In contrast, exploring the path of travel to reduce anxiety through psychological capital may operate through mechanisms such as mastery learning and cognitive flexibility.^[36]^ Successfully planning and adapting to a new environment provides a concrete, manageable mastery experience. These experiences may challenge and negate the general feeling of powerlessness and may help enhance self-efficacy, which is a core component of psychological capital.^[37]^ In addition, exposure to novelty may require and train cognitive flexibility and problem-solving, which may be generalized to the ability to deal with daily stress and thus may be associated with lower anxiety.^[38]^ The anticipation phase before travel may also support hope by directing attention to future positive events, which is another aspect of psychological capital.^[39]^

### 4.2. Methodological rigor and contextualization of observed mechanisms

The study design enhances the credibility of the proposed mechanisms, but their interpretation needs to be done with caution within methodological limitations. Compared with cross-sectional studies, the prospective design used in this study has a time gap between exposure factors and outcome measures, which provides a stronger basis for inferring causality and reduces the possibility of reverse causality of “current anxiety inhibits exploration motivation.”^[40]^ Sequential adjustment models show that socioeconomic factors explain a significant portion of the initial association, a key finding that highlights a major mechanism: social stratification of opportunity.^[41]^ The ability to undertake autonomous, motivated travel is itself a resource shaped by deep social causes. Therefore, it is likely that part of the observed “effect” is attributable to upstream social determinants that both contribute to travel behavior and promote better mental health.^[42]^

However, after adjusting for these and other confounding factors, the independent association remained, suggesting that the quality of experience of travel behavior itself may have added value. This residual association is what our proposed cognitive-affective mechanism is trying to explain. The subgroup analysis found that the effect was stronger in the psychosocially vulnerable population, which provides an important perspective. One mechanistic explanation is stress buffering: for individuals with low social support, travel may provide compensatory experiences of mastery and social contact, which may help build psychological capital in the absence of traditional buffering resources. This interpretation is consistent with stress-buffering models observed in tourism contexts.^[43]^ Another mechanism that is not mutually exclusive involves differential susceptibility; that is, certain individuals with unmeasured traits are both more likely to travel in adversity and more likely to derive mental health benefits from travel.^[44]^ Therefore, the mediating pathways we observed may partly reflect that existing psychological resources can not only promote exploratory behavior but also be directly associated with lower anxiety. Nonetheless, the travel experience itself may further reinforce these resources in a positive feedback loop.^[45]^ Future intervention research is needed to clarify this dynamic.

### 4.3. Contrast with prior research and mechanism-specific context

Our findings require a more nuanced interpretation of prior epidemiological evidence. They consolidate a large body of literature linking leisure activities to better mental health, but at the same time provide a critical refinement of the literature by pointing out that “aggregated measures may mask the opposite or null effects of different activity subtypes.”^[46]^ A study that found a weak overall association between “travel frequency and “well-being may have missed the stronger association of exploratory travel on anxiety, which was diluted by the null effect of other types of travel on the outcome. Our work, along with other recent studies that have differentiated between types of activity, has shifted the field from a focus on “dose to a focus on “function aligned with mechanism.”^[47]^

The cultural context of our Chinese cohort may influence the salience and the way in which the proposed mechanism operates. The strong association between nostalgic travel and the meaning of life may be amplified in the context of southern Chinese culture due to the emphasis on filial piety, ancestral connection, and place-based identity.^[48]^ On the contrary, in a cultural environment with more emphasis on individualism, it may be more prominent to explore the mechanism of influencing the meaning of life through self-expansion.^[49]^ This cross-cultural difference is an important area for future comparative research.

### 4.4. Implications for an aging society

This study shifts the focus from “outreach activities per se” to “fostering meaningful engagement that is aligned with individual motivations and psychological needs,” thereby extending to the realm of practice. For clinical and public health practice, it argues for the inclusion of simple leisure motivation assessments in geriatric care to guide individualized activity recommendations.^[50]^ For the community planning and tourism industry, it highlights the opportunity to develop tourism products that are easy to participate in and meet the needs of both continuity and growth.^[51]^ From a policy perspective, promoting such flows, which are psychologically rich in later life, should be seen as an investment in the psychological capital of an aging population.^[52]^

### 4.5. Limitations and future directions

In considering these mechanistic explanations, several limitations inherent to observational life course epidemiological studies must be acknowledged. First, and most importantly for causal inference, baseline anxiety and meaning in life were not assessed. Although exposures and outcomes were measured prospectively, the analyses estimate follow-up outcome levels rather than within-person change. In addition, the proposed mediating variables (self-continuity, psychological capital) were measured at the same point in time as the exposures, which limits our ability to establish a chronological order in the chain of mediators. These relationships may reflect stable traits or reverse causation. Second, there may still be residual confounding by unmeasured factors that do not change over time (e.g., innate personality, such as openness, extraversion; or physical activity/function at baseline), which may bias the observed association. Third, our measures, although validated, do not capture all of the qualitative details of the travel experience that may mediate the proposed mechanisms. Fourth, our sample was derived from a larger cohort after applying exclusion criteria (see Fig. 1) and may be healthier and of higher socioeconomic status than the general elderly population, which may limit the universality of the findings. If the ability to travel is a sign of some potential advantage that is not fully captured by the covariates, then this sample selection may lead to an overestimation of the benefit. Related to this, missing data, although treated with multiple imputation, may still introduce bias if the missing mechanism is not random. Finally, although a false-discovery rate correction was applied to the primary outcome, a large number of statistical tests were performed in subgroup and mediation analyses, which increased the risk of type I errors (false positives), so the results, particularly the interaction effects, should be considered “hypothesis-generating” in nature.

These limitations guide future studies to adopt study designs that are more suitable for testing mechanisms. We need to develop studies with multiple repeated measures of motivation, proposed mediating variables, and outcomes to model temporal dynamics and strengthen causal inferences about mediating roles. Qualitative and mixed-methods research is essential to empirically lay out and elaborate the proposed cognitive and affective processes from a participant perspective. Most importantly, these observational findings provide a strong rationale for theory-based intervention trials. The next step is to design and evaluate “targeted activity prescriptions,” that is, to examine whether interventions that specifically foster recall or mastery experiences through structured activities can lead to differential improvements in life meaning and anxiety as predicted by our model. Future studies should also strive to incorporate more comprehensive measures of potential confounders, e.g., personality and objective physical function, and employ methods such as instrumental variables or fixed effects models within individuals to better account for unobserved heterogeneity.

## 5. Conclusion

This prospective cohort study suggests that the psychological associations of leisure travel in older adults are not uniform but are significantly influenced by underlying motivation. Exploration-driven travel was prospectively associated with lower levels of anxiety, partly statistically mediated by psychological capital, while nostalgia-driven travel was specifically associated with a greater sense of meaning in life, partly statistically mediated by self-continuity. Those with a combination of the 2 motivations, as well as those with psychosocial vulnerabilities, showed the strongest associations. However, these associations were observed within the limitations of observational studies and may be influenced by residual confounders and social stratification of travel opportunities. These findings suggest that the value of an activity for mental health lies not only in its frequency but also in whether its function is compatible with specific psychological needs and resources. Although this study is observational and may be influenced by confounding factors, the results advocate a more detailed and motivational approach in research and practice to use leisure activities to promote multi-dimensional mental health in the elderly population, and point out promising directions for targeted interventions and people-oriented public health strategies.

## Author contributions

**Data curation:** Ruyue Li, Nana Zhao.

**Investigation:** Ruyue Li, Nana Zhao.

**Methodology:** Ruyue Li, Nana Zhao.

**Software:** Ruyue Li.

**Validation:** Ruyue Li.

**Writing – original draft:** Ruyue Li, Nana Zhao.

**Writing – review & editing:** Ruyue Li, Nana Zhao.



## References

[1] Venkatapuram S Amuthavalli Thiyagarajan J. The capability approach and the WHO healthy ageing framework (for the UN decade of healthy ageing). Age Ageing. 2023;52(Suppl 4):iv6–9. 10.1093/ageing/afad126 PMC10615052 37902511

[2] Sunderland N Stevens F Knudsen K Cooper R Wobcke M. Trauma aware and anti-oppressive arts-health and community arts practice: guiding principles for facilitating healing, health and wellbeing. Trauma Violence Abuse. 2023;24:2429–47. 10.1177/15248380221097442 35577560

[3] Yap YY Tan SH Tan BC Tan SK. Smart tourism technologies and tourist satisfaction: a systematic literature review and research agenda. Acta Psychol (Amst). 2025;258:105191. 10.1016/j.actpsy.2025.105191 40633450

[4] Lindsay-Smith G Pyke J Gamage A Nguyen V de Lacy T. Tourism operator mental health and its relationship with SME organisational resilience during disasters. Tour Manag Perspect. 2022;42:100961. 10.1016/j.tmp.2022.100961 PMC9576215 36277033

[5] Bayih BE Singh A. Modeling domestic tourism: motivations, satisfaction and tourist behavioral intentions. Heliyon. 2020;6:e04839. 10.1016/j.heliyon.2020.e04839 PMC7505762 32995592

[6] Carstensen LL. Socioemotional selectivity theory: the role of perceived endings in human motivation. Gerontologist. 2021;61:1188–96. 10.1093/geront/gnab116 PMC8599276 34718558

[7] Sedikides C Wildschut T. Nostalgia as motivation. Curr Opin Psychol. 2023;49:101537. 10.1016/j.copsyc.2022.101537 36577228

[8] Liu Z Tian N Guo Q Dai Y Tang H Liu C. Psychometric properties of the three-dimensional meaning in life scale in Chinese undergraduate students. Sci Rep. 2025;15:30995. 10.1038/s41598-025-16656-4 PMC12374993 40849331

[9] Hughes EK Slotter EB Lewandowski GW Jr. Expanding who I am: validating the self-expansion preference scale. J Pers Assess. 2020;102:792–803. 10.1080/00223891.2019.1641109 31355680

[10] Goldman-Gerber V Schwartz I Rand D. Upper extremity self-efficacy correlates with daily hand-use of individuals with high functional capacity post-stroke. Disabil Rehabil. 2023;45:2301–6. 10.1080/09638288.2022.2087764 35722769

[11] Kuşcu Karatepe H Tiryaki Şen H Türkmen E. Predicting work performance and life satisfaction of nurses and physicians: the mediating role of social capital on self-efficacy and psychological resilience. Perspect Psychiatr Care. 2022;58:2542–51. 10.1111/ppc.13092 35430728

[12] Wora N Pachana NA Taephant N. Leisure activity and subjective well-being of older adults in Australia and Thailand: the impact of affiliation, restoration and meaning. Australas J Ageing. 2025;44:e13402. 10.1111/ajag.13402 39787299

[13] Nimrod G Shrira A. The paradox of leisure in later life. J Gerontol B Psychol Sci Soc Sci. 2016;71:106–11. 10.1093/geronb/gbu143 PMC4861252 25315158

[14] Wang Y He X Zhang F Zhang X Hu X Xie X. The influence of progeny-parents family travel on the well-being of the elderly in filial piety culture. PLoS One. 2024;19:e0299565. 10.1371/journal.pone.0299565 PMC11081327 38722872

[15] Dodell-Feder D Shovestul B Woodyatt J Popov V Germine L. Social anhedonia, social networks, and psychotic-like experiences: a test of social deafferentation. Psychiatry Res. 2020;284:112682. 10.1016/j.psychres.2019.112682 PMC7012747 31735375

[16] Gyasi RM Mariwah S Boateng S. ‘The new geriatric giants’: how do loneliness and social isolation contribute to probable depression in older adults? J Biosoc Sci. 2025;57:549–61. 10.1017/S0021932025100400 40827035

[17] Greenland S. For and against methodologies: some perspectives on recent causal and statistical inference debates. Eur J Epidemiol. 2017;32:3–20. 10.1007/s10654-017-0230-6 28220361

[18] Dai YY Shie AJ Chu JH Wu YJ. Low-carbon travel motivation and constraint: scales development and validation. Int J Environ Res Public Health. 2022;19:5123. 10.3390/ijerph19095123 PMC9105957 35564516

[19] Nylund-Gibson K Garber AC Carter DB. Ten frequently asked questions about latent transition analysis. Psychol Methods. 2023;28:284–300. 10.1037/met0000486 35834194

[20] Zhang C Wang T Zeng P. Reliability, validity, and measurement invariance of the general anxiety disorder scale among Chinese Medical University students. Front Psychiatry. 2021;12:648755. 10.3389/fpsyt.2021.648755 PMC8170102 34093269

[21] Xia HZ Gao L Wang Y Song H Shi BX. Development of the Chinese version of meaning in life scale for cancer patients and psychometric evaluation. J Clin Nurs. 2017;26:3298–304. 10.1111/jocn.13675 27878884

[22] Ji LJ Imtiaz F Su Y Zhang Z Bowie AC Chang B. Culture, aging, self-continuity, and life satisfaction. J Happiness Stud. 2022;23:3843–64. 10.1007/s10902-022-00568-5 PMC9514170 36187718

[23] Snyder CR Harris C Anderson JR. The will and the ways: development and validation of an individual-differences measure of hope. J Pers Soc Psychol. 1991;60:570–85. 10.1037//0022-3514.60.4.570 2037968

[24] Connor KM Davidson JR. Development of a new resilience scale: the Connor-Davidson Resilience Scale (CD-RISC). Depress Anxiety. 2003;18:76–82. 10.1002/da.10113 12964174

[25] Bień A Niewiadomska I Korżyńska-Piętas M. General self-efficacy as a moderator between severity of menopausal symptoms and satisfaction with life in menopausal women. Front Public Health. 2024;12:1426191. 10.3389/fpubh.2024.1426191 PMC11390549 39267631

[26] Prasath PR Xiong Y Zhang Q Jeon L. Psychological capital, well-being, and distress of international students. Int J Adv Couns. 2022;44:529–49. 10.1007/s10447-022-09473-1 PMC9199344 35730062

[27] Khan MM Taylor S Morry C Sriram S Demir I Siddiqi M. How reliable is the asset score in measuring socioeconomic status? Comparing asset ownership reported by male and female heads of households. PLoS One. 2023;18:e0279599. 10.1371/journal.pone.0279599 PMC9955958 36827269

[28] He F Zhou Q Zhao Z Zhang Y Guan H. Effect of perceived social support and dispositional optimism on the depression of burn patients. J Health Psychol. 2016;21:1119–25. 10.1177/1359105314546776 25161173

[29] Asokan GV Awadhalla M Albalushi A. The magnitude and correlates of geriatric depression using Geriatric Depression Scale (GDS-15) - a Bahrain perspective for the WHO 2017 campaign ‘Depression - let’s talk’. Perspect Public Health. 2019;139:79–87. 10.1177/1757913918787844 29993323

[30] Monserud MA. Leisure activities and depressive symptoms among older men and women in Mexico: implications of physical health. J Aging Health. 2023;35:94–107. 10.1177/08982643221108036 PMC9747986 35698826

[31] Tian HB Qiu YJ Lin YQ Zhou WT Fan CY. The role of leisure satisfaction in serious leisure and subjective well-being: evidence from Chinese marathon runners. Front Psychol. 2020;11:581908. 10.3389/fpsyg.2020.581908 PMC7720892 33329237

[32] Abeyta AA Pillarisetty S. Nostalgia supports a meaningful life. Curr Opin Psychol. 2023;49:101520. 10.1016/j.copsyc.2022.101520 36549093

[33] Barry TJ Hallford DJ. Transdiagnostic and transtherapeutic strategies for optimising autobiographical memory. Behav Res Ther. 2024;180:104575. 10.1016/j.brat.2024.104575 38852230

[34] Golovchanova N Owiredua C Boersma K Andershed H Hellfeldt K. Presence of meaning in life in older men and women: the role of dimensions of frailty and social support. Front Psychol. 2021;12:730724. 10.3389/fpsyg.2021.730724 PMC8448055 34539530

[35] Lund K Argentzell E Leufstadius C Tjörnstrand C Eklund M. Joining, belonging, and re-valuing: a process of meaning-making through group participation in a mental health lifestyle intervention. Scand J Occup Ther. 2019;26:55–68. 10.1080/11038128.2017.1409266 29179630

[36] Terhorst Y Baumeister H McCracken LM Lin J. Further development in the assessment of psychological flexibility: validation of the German committed action questionnaire. Health Qual Life Outcomes. 2020;18:260. 10.1186/s12955-020-01497-8 PMC7397648 32746838

[37] Zhao Y Niu J Wang Y Wang P. Assessment of the psychometric properties of the perceived empathic and social self-efficacy scale in Chinese adolescents. Psychol Res Behav Manag. 2024;17:3677–88. 10.2147/PRBM.S484649 PMC11514698 39469225

[38] Ebner NC Horta M El-Shafie D. New directions for studying the aging social-cognitive brain. Curr Opin Psychol. 2024;56:101768. 10.1016/j.copsyc.2023.101768 PMC10939782 38104362

[39] Gallagher MW Long LJ Richardson A. Examining hope as a transdiagnostic mechanism of change across anxiety disorders and CBT treatment protocols. Behav Ther. 2020;51:190–202. 10.1016/j.beth.2019.06.001 PMC7000132 32005336

[40] Munafò MR Davey Smith G. Robust research needs many lines of evidence. Nature. 2018;553:399–401. 10.1038/d41586-018-01023-3 29368721

[41] Hsieh YC Apostolopoulos Y Hatzudis K Sönmez S. Social, occupational, and spatial exposures and mental health disparities of working-class Latinas in the US. J Immigr Minor Health. 2016;18:589–99. 10.1007/s10903-015-0231-z 26044667

[42] Amroussia N Gustafsson PE Mosquera PA. Explaining mental health inequalities in Northern Sweden: a decomposition analysis. Glob Health Action. 2017;10:1305814. 10.1080/16549716.2017.1305814 PMC5496092 28562191

[43] Liu H Tan Q Mai H. Stress-buffering effects of social support on tourism employees during the COVID-19 pandemic: a moderated mediation model. Int J Environ Res Public Health. 2023;20:2342. 10.3390/ijerph20032342 PMC9915072 36767707

[44] Markovitch N Knafo-Noam A. Sensitivity, but to which environment? Individual differences in sensitivity to parents and peers show domain-specific patterns and a negative genetic correlation. Dev Sci. 2021;24:e13136. 10.1111/desc.13136 34155726

[45] Neveu JP Bégout P. Measuring motivational patterns: a formal approach of conservation of resources theory. Nonlinear Dynamics Psychol Life Sci. 2025;29:431–51. 40579805

[46] Olaru G Laukka EJ Dekhtyar S Sarwary A Brehmer Y. Association between personality traits, leisure activities, and cognitive levels and decline across 12 years in older adults. Psychol Aging. 2023;38:277–90. 10.1037/pag0000743 37036694

[47] Fancourt D Steptoe A. The art of life and death: 14 year follow-up analyses of associations between arts engagement and mortality in the English Longitudinal Study of Ageing. BMJ. 2019;367:l6377. 10.1136/bmj.l6377 PMC7190042 31852659

[48] Xiao C Patrician PA Montgomery AP Wang Y Jablonski R Markaki A. Filial piety and older adult caregiving among Chinese and Chinese-American families in the United States: a concept analysis. BMC Nurs. 2024;23:115. 10.1186/s12912-024-01789-0 PMC10863110 38347512

[49] Oishi S Westgate EC. A psychologically rich life: beyond happiness and meaning. Psychol Rev. 2022;129:790–811. 10.1037/rev0000317 34383524

[50] Fakoya OA McCorry NK Donnelly M. Loneliness and social isolation interventions for older adults: a scoping review of reviews. BMC Public Health. 2020;20:129. 10.1186/s12889-020-8251-6 PMC7020371 32054474

[51] Tuo J Deng R Tao M. Cultural factors and senior tourism: evidence from the Chinese context. Front Psychol. 2022;13:1030059. 10.3389/fpsyg.2022.1030059 PMC9731214 36507043

[52] Chen Q Chi Q Chen Y Lyulyov O Pimonenko T. Does population aging impact China’s economic growth? Int J Environ Res Public Health. 2022;19:12171. 10.3390/ijerph191912171 PMC9564793 36231472

