# Supplementary material for: The differential associations of reminiscence and exploratory travel with late-life psychological well-being: A prospective cohort study
Source: Medicine (Baltimore). 2026 Oct 16;105(42):e51140. doi: 10.1097/MD.0000000000051140 (PMC13681161; doi:10.1097/MD.0000000000051140)
Supplement: Supplementary file 1 [file medi-105-e51140-s001.docx]

| **Supplementary Table S1. Fit indices for latent class solutions (1–5 classes)** | | | | | | | |
| --- | --- | --- | --- | --- | --- | --- | --- |
| **Model** | **AIC** | **BIC** | **aBIC** | **Entropy** | **LMR-LRT P** | **BLRT P** | **Smallest class, %** |
| **1-class** | 13620.5 | 13680.2 | 13640.1 | — | — | — | 100.0 |
| **2-class** | 13020.7 | 13110.4 | 13050.2 | 0.78 | <0.001 | <0.001 | 32.5 |
| **3-class** | 12680.3 | 12800.1 | 12720.5 | 0.82 | 0.003 | <0.001 | 18.7 |
| **4-class** | 12420.8 | 12500.3 | 12420.7 | 0.85 | 0.012 | <0.001 | 20.4 |
| **5-class** | 12390.6 | 12580.2 | 12490.5 | 0.79 | 0.342 | 0.078 | 8.2 |
| Note: AIC, Akaike information criterion; BIC, Bayesian information criterion; aBIC, sample-size adjusted BIC; LMR-LRT, Lo-Mendell-Rubin adjusted likelihood ratio test; BLRT, bootstrap likelihood ratio test. LMR-LRT and BLRT compare the k-class model with the (k−1)-class model; a significant P value indicates that the k-class model fits better. Entropy values >0.80 indicate good classification quality. The 4-class solution was selected because it had the lowest BIC and aBIC, acceptable entropy, significant LMR-LRT and BLRT, and the most interpretable class structure. The 5-class solution did not improve model fit and produced a small class (8.2%), so it was not retained. | | | | | | | |
